# 434. Impact of a Blood Culture Stewardship Initiative Among Pediatric Febrile Neutropenia Patients on Reducing Carbon Emissions

**DOI:** 10.1093/ofid/ofaf695.146

**Published:** 2026-01-11

**Authors:** Adriana Gardner, Michelle L Kussin, Carmen Dunn Smith, Muayad Allali

**Affiliations:** Purdue University College of Pharmacy, Los Altos, CA; IU Health, Indianapolis, Indiana; Indiana University Health, Danville, Indiana; Indiana University School of Medicine, Indianapolis, Indiana

## Abstract

**Background:**

In the United States, the healthcare sector is responsible for about 8.5% of national greenhouse gas emissions. Diagnostic stewardship initiatives can help mitigate these emissions. At Riley Children’s Hospital, a blood culture stewardship initiative was implemented to reduce the number of repeat blood cultures ordered for pediatric febrile neutropenia patients. The purpose of this study was to calculate the reduction in carbon emissions from the blood culture microbiology waste associated with the blood culture stewardship initiative.

**Figure:**
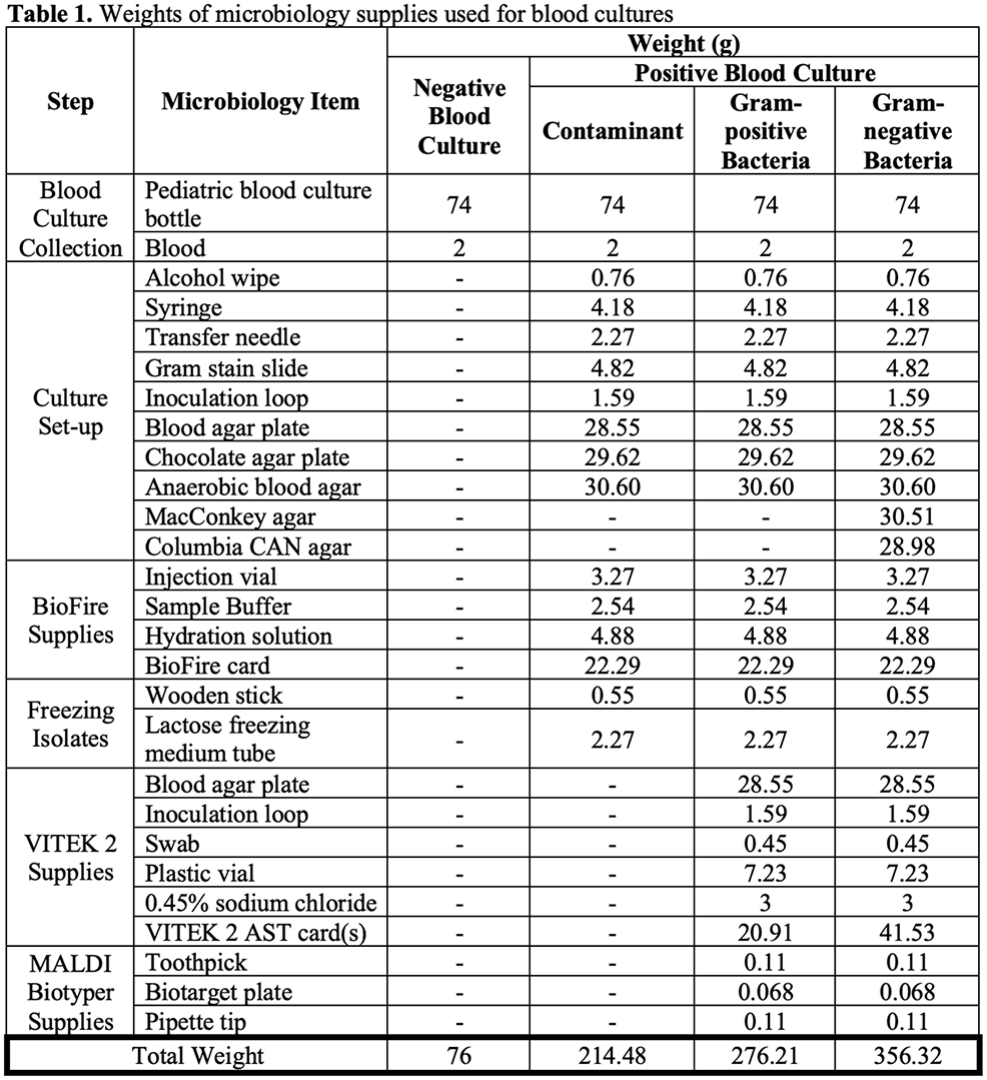

**Figure:**
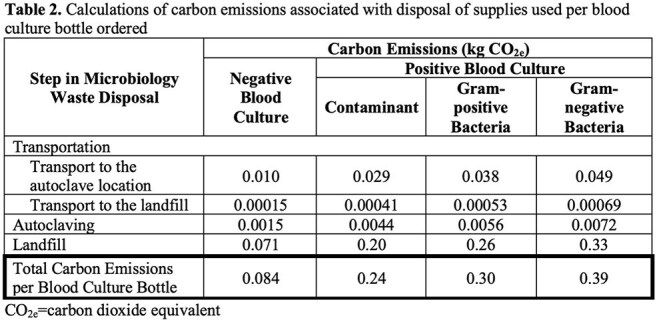

**Methods:**

Starting January 1, 2023, a new febrile neutropenia blood culture protocol was implemented, limiting repeat blood cultures after 48 hours to patients with hemodynamic instability or recurrent fever (defined as a new fever occurring ≥72 hours after being afebrile). The number of blood culture bottles use before (January 2017 to December 2022) and after (January 2023 to December 2023) the initiative were calculated. Then microbiology supplies used for negative and positive blood cultures were documented and weighed. The Mazzetti M+Wastecare calculator was used to calculate the carbon emissions from the transportation, autoclaving, and landfill disposal of the supplies.

**Figure:**
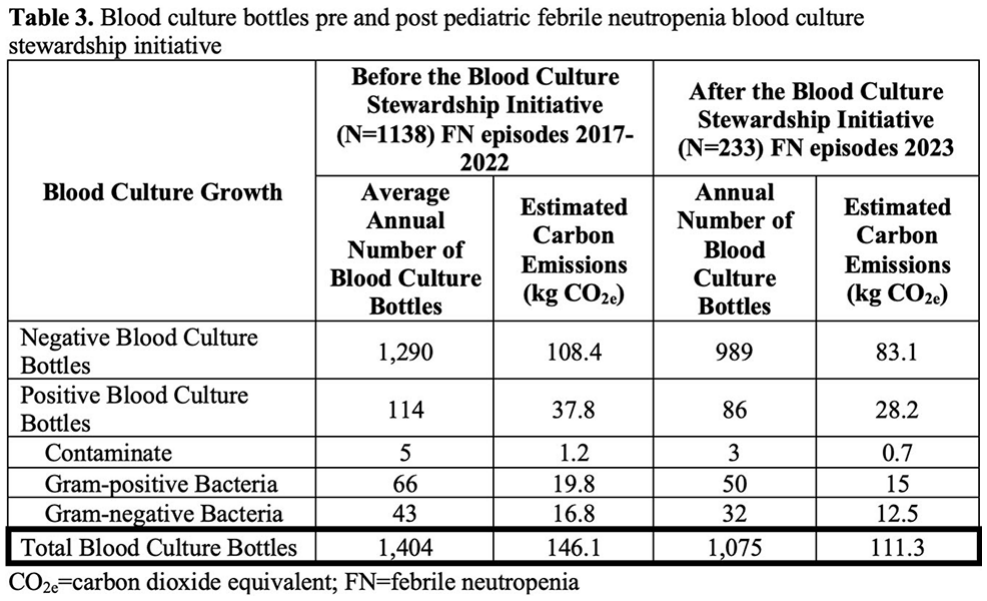

**Results:**

Before and after implementation of the blood culture stewardship initiative, 1,403 bottles per year and 1,075 bottles per year were utilized respectively, resulting in a 31% reduction in use annually. Weights of supplies used for negative and positive blood cultures were recorded (Table 1). Calculated carbon emissions associated with the weights of the supplies used for positive and negative cultures are displayed in Table 2. Overall, the blood culture stewardship intervention reduced carbon emissions by 34.9 kg CO_2e_ annually, equivalent to driving approximately 87 miles in an average passenger vehicle (Table 3).

**Conclusion:**

The blood culture stewardship initiative among pediatric febrile neutropenia patients resulted in a positive environmental impact as it decreased carbon emissions equivalent to driving approximately 87 miles. Given that carbon emissions associated with manufacturing, packaging, and delivery of supplies were not factored in, this is an underestimate of the full annual reduction in carbon emissions.

**Disclosures:**

All Authors: No reported disclosures

